# Supplementary material for: Molecular Characterization of Astrocytoma Progression Towards Secondary Glioblastomas Utilizing Patient-Matched Tumor Pairs
Source: Cancers (Basel). 2020 Jun 26;12(6):1696. doi: 10.3390/cancers12061696 (PMC7352509; doi:10.3390/cancers12061696)
Supplement: Supplementary file 1 [file cancers-12-01696-s001.zip › Supporting_Tables_and_Figures/Figure_S2.pdf]

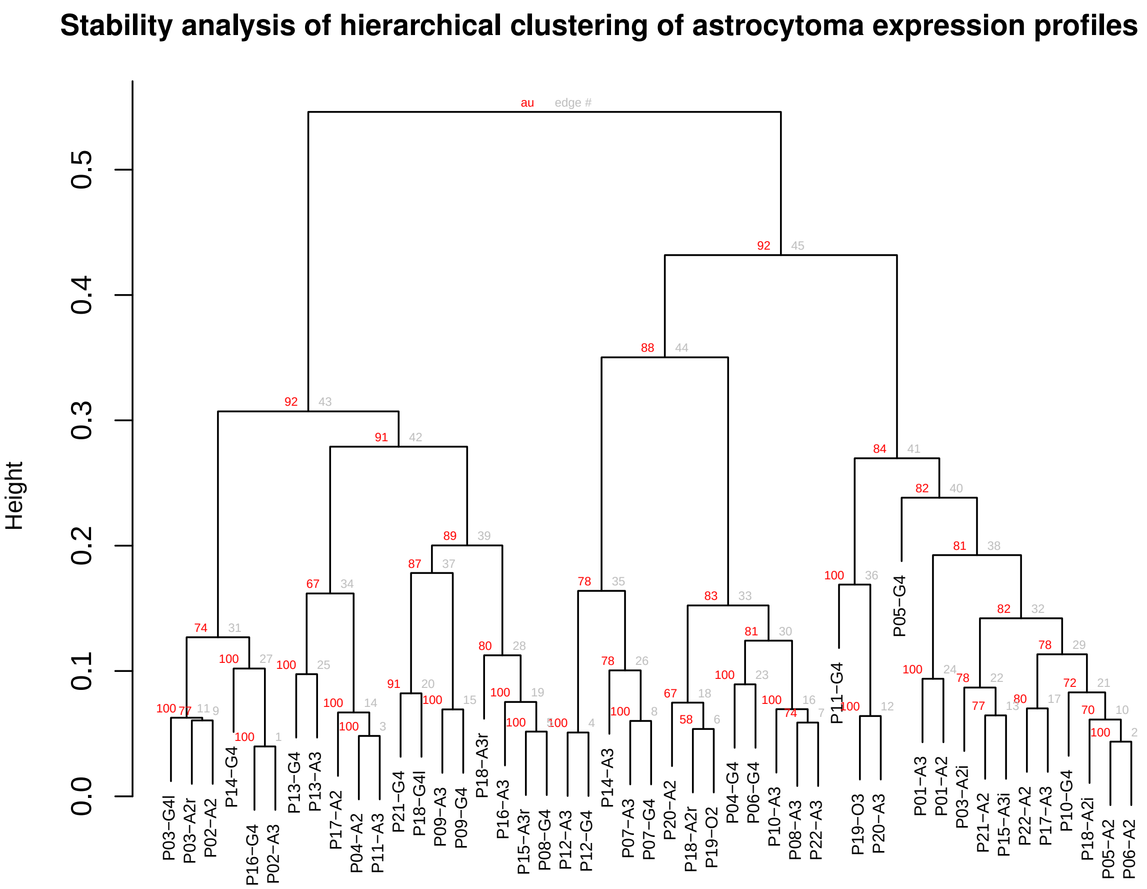

Figure S2. Stability analysis of the hierarchical clustering of astrocytoma gene expression profiles initially shown as column-dendrogram in Figure 3a. The R-package pvclust was used with standard bootstrap settings to analyze the stability of the clusters. Approximately unbiased bootstrap values (AU values) are shown in red and grey values specify the rank of the cluster during creation. Validity of a clusters is the better the closer its AU value is to 100. The three major gene expression clusters show good stabilities with AU values from 84 to 92 with respect to the limitations of our small data set along with relatively heterogeneous gene expression profiles. All patient-matched initial and recurrent tumors that directly clustered together have AU values of 100.
