# Supplementary material for: Molecular Characterization of Astrocytoma Progression Towards Secondary Glioblastomas Utilizing Patient-Matched Tumor Pairs
Source: Cancers (Basel). 2020 Jun 26;12(6):1696. doi: 10.3390/cancers12061696 (PMC7352509; doi:10.3390/cancers12061696)
Supplement: Supplementary file 1 [file cancers-12-01696-s001.zip › Supporting_Tables_and_Figures/Figure_S3.pdf]

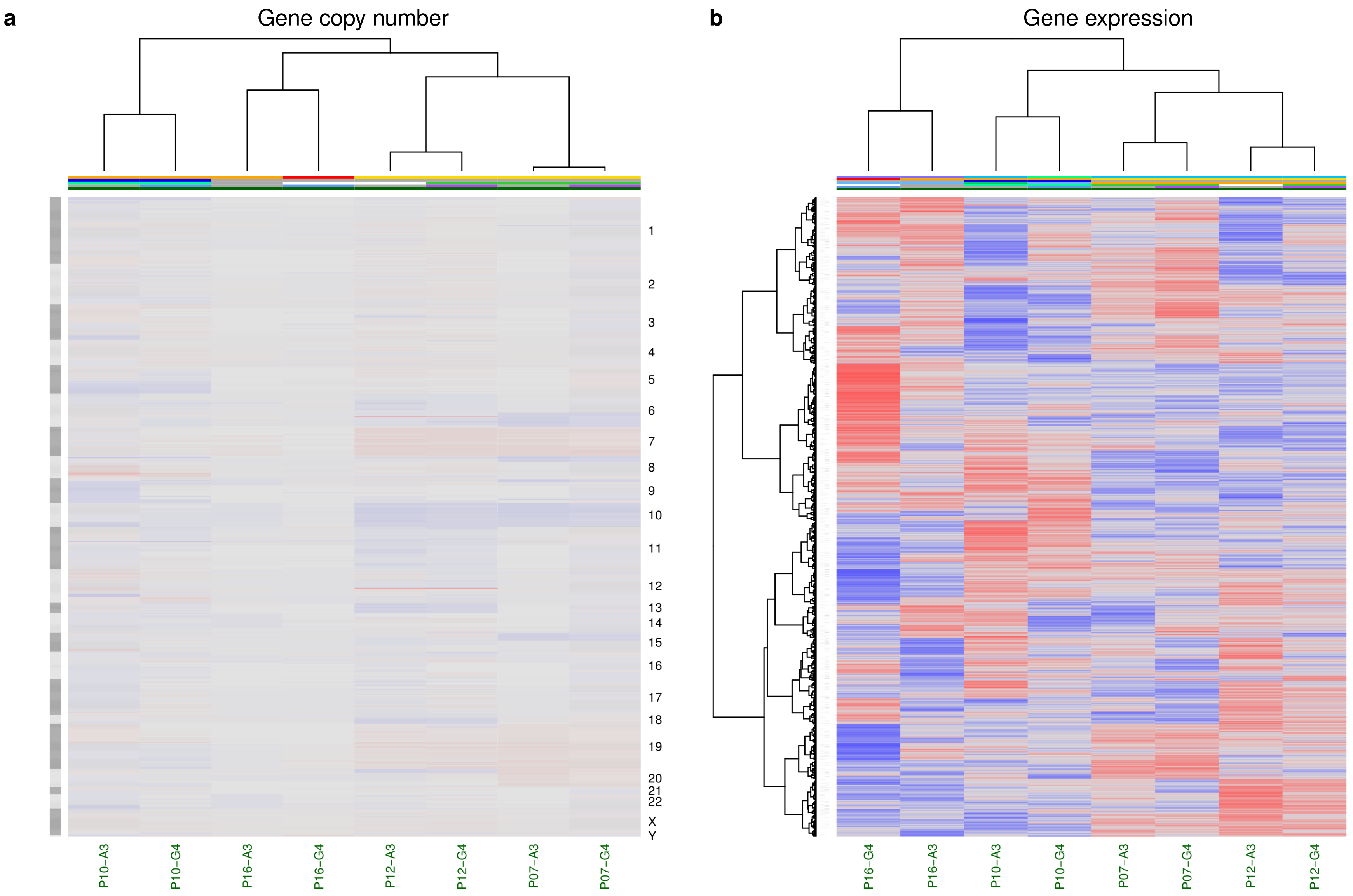

Figure S3. Genome-wide heatmaps of gene copy number and expression profiles of astrocytomas patients that have been treated by irradiation and chemotherapy after the resection of their initial tumor. Tumors were clustered based on the similarity of their molecular alteration profiles (columns) and their corresponding gene-specific values are visualized (rows). **a,** The gene copy number heatmap represents gene deletions (blue regions), gene duplications (red regions) and genes with unchanged copy number (grey regions) in tumor compared to normal DNA. The organization of this heatmap follows the description provided in the caption of Figure 2a. **b,** The gene expression heatmap represents z-score scaled expression levels of each gene with values clearly less than zero in blue shades, values about zero in grey, and values clearly greater than zero in red shades. The organization of this heatmap follows the description provided in the caption of Figure 3a.
